# Genotype and clinical phenotype characteristics of *MAX* germline mutation–associated pheochromocytoma/paraganglioma syndrome

**DOI:** 10.3389/fendo.2024.1442691

**Published:** 2024-08-30

**Authors:** Bijun Lian, Jun Lu, Xudong Fang, Yiming Zhang, Wei Wang, Yi He, Hongyuan Yu, Feiping Li, Junwei Wang, Weiying Chen, Xiaoping Qi

**Affiliations:** ^1^ Laboratory Department of Oncologic and Urologic Surgery, The 903rd PLA Hospital, Hangzhou Medical College, Hangzhou, Zhejiang, China; ^2^ Department of Urology, Changhai Hospital, Navy Military Medical University, Shanghai, China; ^3^ Department of Urology, Taizhou Hospital of Zhejiang Province Affiliated to Wenzhou Medical University, Enze Hospital of Hangzhou Medical College, Taizhou Enze Medical Center (Group), Taizhou, China; ^4^ Department of Urology, Tiantai People’s Hospital of Zhejiang Province, Taizhou, Zhejiang, China; ^5^ Department of Urology, The First Hospital of Jiaxing, The First Affiliated Hospital of Jiaxing University, Jiaxing, Zhejiang, China

**Keywords:** multiple endocrine neoplasia, pheochromocytoma, paraganglioma, genealogy, MAX gene

## Abstract

**Objective:**

The aim of this study was to investigate the genotypic and clinical phenotypic characteristics of *MAX* germline mutation–associated pheochromocytoma (PCC) and paraganglioma (PGL).

**Methods:**

We retrospectively analyzed the family investigation data and clinical genetic characteristics of six individuals from three independent families with PCC carrying *MAX* germline mutations from December 2005 to March 2024. A literature review was then conducted of the six carriers and another 103 carriers from the other 84 families with *MAX* germline mutations reported previously.

**Results:**

There were 109 patients in 87 families with all five exons and 53 types of *MAX* germline mutations. p.R33* (c.97C>T; 21.1%), p.R75* (c.223C>T; 13.8%), and p.A67D (c.200C>A; 7.3%), which accounted for 42.2% of mutations detected, were the most common mutations. Moreover, 101 (92.7%) patients developed PCCs, including 59 bilateral PCCs and 42 unilateral PCCs, and 19 (18.8%) patients showed metastasis. The mean age at diagnosis was 32.8 ± 12.6 (13-80) years. The male-to-female ratio was 1.3:1. In 11 (10.9%) patients, the PCC was accompanied by chest or abdominal PGL, and one other patient had sole head and neck PGL. Nine (8.3%) patients also had functional pituitary adenomas, 11 (10.9%) developed other neuroendocrine tumors (NETs), and 7 (6.4%) presented with concomitant non-NET. Meanwhile, *MAX*-p.Q82Tfs*89 and p.E158A mutations are reported for the first time in this study.

**Conclusion:**

*MAX* germline mutations may cause new types of multiple endocrine neoplasia. A comprehensive baseline assessment of neural crest cell–derived diseases is recommended for all individuals with *MAX* germline mutations. The risk of bilateral and metastatic PCCs should also be considered.

## 1 Introduction

Pheochromocytoma (PCC) and paraganglioma (PGL) are collectively referred to as PPGL and they originate from the neural crest cells of the embryonic ectoderm. PPGL is a rare neuroendocrine tumor (NET) that often has endocrine functions (1, 2). Approximately 40% of PPGLs manifest as genetic syndromes involving at least 24 characteristic pathogenic germline driving genes (1–5). These gene mutations are divided into three main clusters based on the activation of a particular signaling pathway. Cluster 1 (C1, i.e., pseudohypoxia signaling cluster) would be implemented with *VHL, FH, SDHA~D, SDHAF2, MDH2, EGLN2/PHD2, IDH1/2/3B, HIF2A/EPAS1, DLST, SUCLG2, SLC25A11* and *IRP1* that controls cellular iron metabolism and negatively regulates HIF2a mRNA translation (not caused by hypoxia). Cluster 2 (C2, i.e., kinase signaling cluster) would mainly include *RET, NF1, TMEM127, MAX, H-RAS, KIF1Bβ, MERTK, MET, MYCN* and *ATRX*, which belongs to the SWI/SNF family of chromatin remodeling proteins, as their upregulation will activate the PI3K/AKT and RAS/MAPK signaling pathways resulting in tumor formation. Finally, cluster 3 (C3, i.e., Wnt signaling cluster) would be implemented with both *CSDE1* and *UBFT* fusion at *MAML3* related to somatic mutations and alterations of any of these genes will result in increase of target genes involved in Wnt receptor and Hedgehog signaling pathways, i.e., the Wnt-altered subtype (5). Classic PPGL-related genetic syndromes include multiple endocrine neoplasia type 2 (MEN 2), von Hippel–Lindau syndrome, neurofibromatosis 1, and PGL1-5, which are caused by pathogenic germline mutations in *RET, VHL, NF1, SDHD, SDHAF2, SDHC, SDHB*, and *SDHA* genes, respectively. In recent years, germline *TMEM127* mutation–related familial PPGL; fumarate hydratase (*FH*) mutation–related hereditary leiomyomatosis and renal cell carcinoma syndrome (HLRCC); and MYC-associated factor X (*MAX*) mutation–related multiple endocrine tumors, including PPGL, have been discovered and reported (1–10). However, the genotype-clinical phenotype correlations of *MAX* mutation–related PPGL are not fully understood. We retrospectively analyzed clinical data from three different families with *MAX* germline mutation–related PCC, and performed a review of the literatures, to discuss the characteristics of *MAX* germline mutations, with the aim of improving the comprehensive ability to diagnose and treat *MAX* mutation–related PPGL.

## 2 Materials and methods

### 2.1 Study population

**Figure 1:**
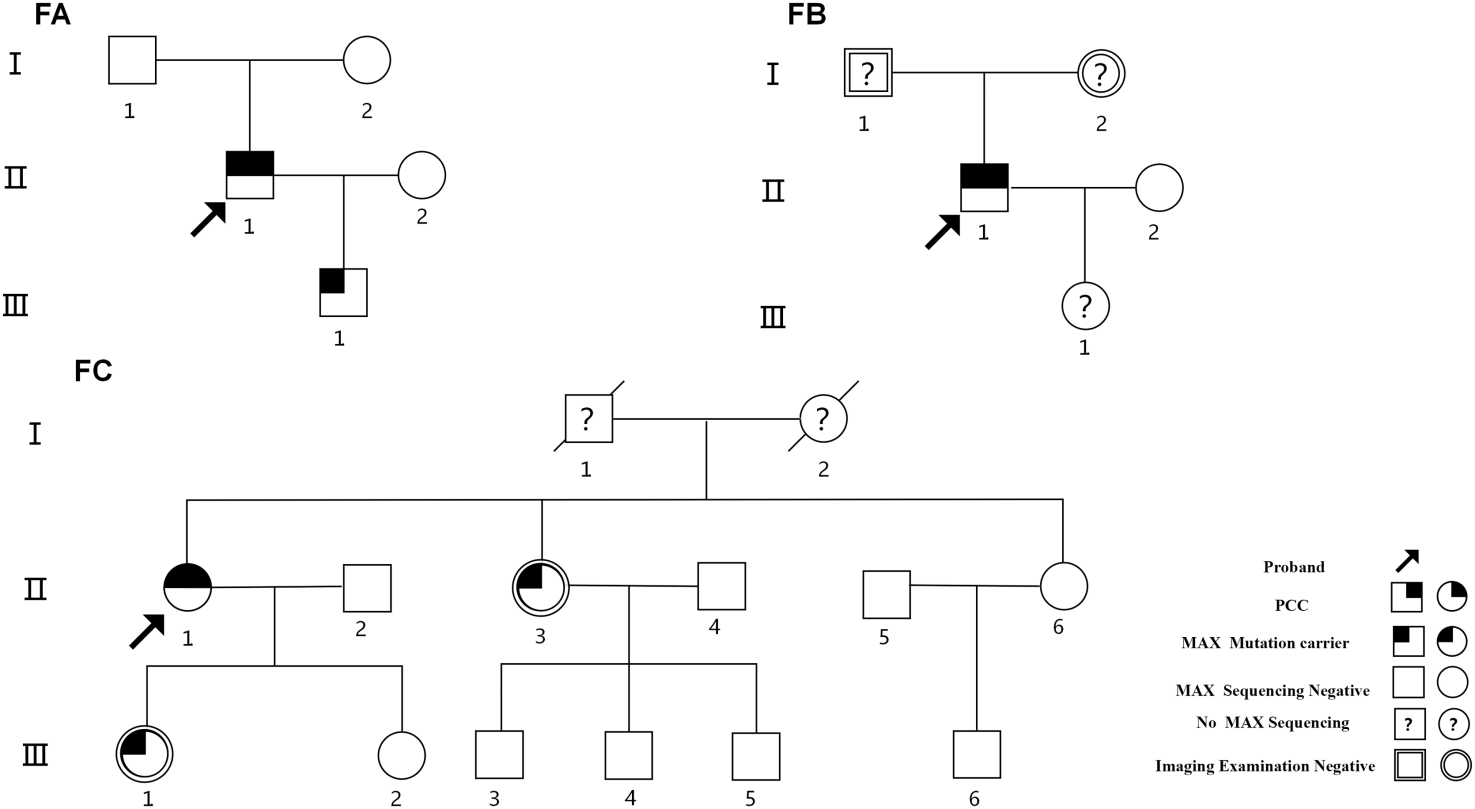
Pedigree investigation of the three *MAX* germline mutation-related families.

Nineteen individuals from three independent PCC pedigree groups diagnosed and treated at the 903rd PLA Hospital, Taizhou Enze Medical Center (Group), People’s Hospital of Tiantai County, and the First Hospital of Jiaxing City from December 2005 to November 2022 were selected for investigation using pedigree analysis (**Figure 1**). Suspected PCC was evaluated based on the plasma-excreted amounts of catecholamines (epinephrine, norepinephrine, and dopamine) and metanephrine/normetanephrine. Other specific biochemical tests, including serum calcium, serum basal calcitonin, parathyroid hormone, growth hormone, prolactin concentrations and so on were tested. Imaging examinations, including Doppler B-ultrasound, computed tomography (CT), and magnetic resonance imaging (MRI) were conducted based on patients’ clinical symptoms, biochemical tests and so on. The diagnosis and metastasis criteria for PPGL were based on the World Health Organization classification of endocrine tumors (1, 2).

### 2.2 Germline genetic testing and protein three-dimensional modeling

Peripheral blood was obtained from 19 individuals in the three PCC pedigree groups for genomic DNA extraction using a QIAamp DNA Blood Midi Kit (Qiagen, Hilden, Germany). Targeted next-generation sequencing of 24 genes associated with hereditary PCC, including *RET, VHL, SDHA-D, SDHAF2, MAX, TMEM127, FH*, and *HIF2A/EPAS1*, was performed. Online software Combined Annotation Dependent Depletion (CADD, https://cadd.gs.washington.edu/snv, accessed on 18 Jan 2024) and Rare Exome Variant Ensemble Learner (REVEL; https://sites.google.com/site/revelgenomics/, accessed on 18 Jan 2024) were used to predict pathogenic variants. Pathogenicity was classified according to the American College of Medical Genetics and Genomics criteria. Sanger sequencing was performed for identification and confirmation. In addition, AlphaFold 2 (V2.3.1) was used to predict the tertiary structures of mutant MAX proteins to validate mutations in specific protein domains.

### 2.3 Literature search strategy and data collection

Two authors independently searched the Cochrane Library, PubMed, Web of Science, and EMBASE up to March 2024. Literature retrieval strategies were as follows: #1 (“MYC associated factor X”[Mesh]) OR *MAX* [Title/Abstract]; #2 (“Pheochromocytoma” [Mesh]) OR Pheochromocytomas [Title/Abstract]); #3 (“Paraganglioma”[Mesh]) OR Paragangliomas [Title/Abstract]) OR Paragangliomata [Title/Abstract]) OR Paragangliomatas [Title/Abstract]; and #4 (#1 and #2 or #3). Full texts of the articles were evaluated for eligibility. Disputes were resolved by consensus among all the authors. A total of 27 publications were identified.

To determine whether there were any genotype-phenotype correlations, we further divided patients with *MAX* mutations into two groups: missense mutation (GM) and non-missense mutation (GN) groups. The GN group included patients with all other types of mutations, such as nonsense, frameshift, alternative splice site, deletion/insertion, duplication, and gene fusion mutations. Clinical parameters, including diagnostic age at presentation and the occurrence rate of PCC, PGL, PA, and other NETs, were compared between patients in the GM and GN groups.

### 2.4 Statistical analysis

SPSS Statistics for Windows, version 25.0 (IBM, Armonk, NY, USA) was used for data processing. Quantitative data were expressed as the mean ± standard deviation, and a Student’s t-test or non-parametric rank sum test was used for intergroup comparisons. The chi-square test or Fisher’s exact probability method was used for intergroup comparisons of categorical data. The significance level was set at *P* < 0.05.

## 3 Results

### 3.1 Clinical features and phenotypic data

#### 3.1.1 Family A

**Figure 2:**
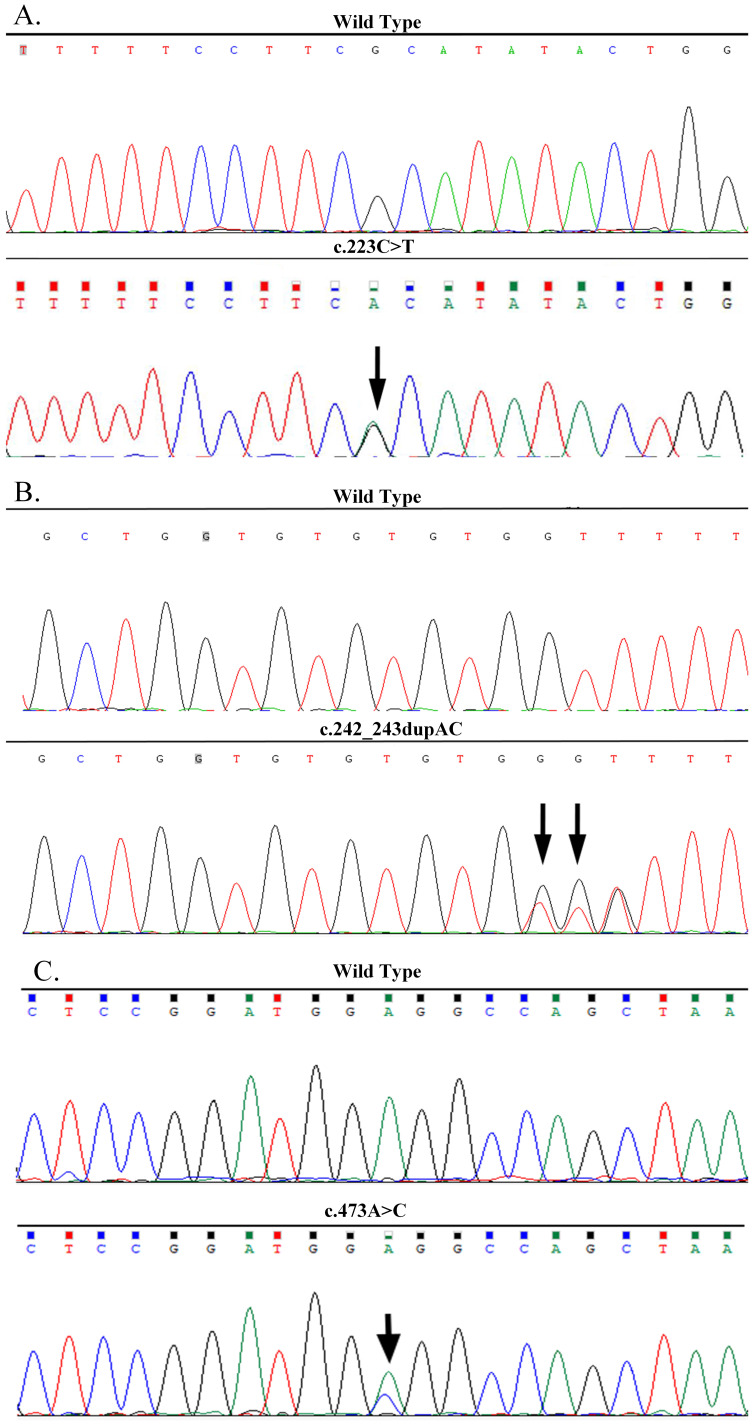
DNA sequencing confirmed the *MAX* mutations in the three families. **(A)** A heterozygous nonsense mutation of p.R75*(c.223C>T) in exon 4 of the *MAX* gene in the proband of Family A. **(B)** A heterozygous frameshift mutation of p.Q82Tfs*89 (c.242_243dupAC) in exon 4 of the *MAX* gene in the proband of Family B. **(C)** A heterozygous missense mutation of p.E158A (c.473A>C) in exon 5 of the *MAX* gene in the proband of Family C.

The proband (**Figures 1**-FA-II-1) was a 24-year-old man who had been diagnosed as having “right PCC” in December 2005 with more than a 2-year history of elevated blood pressure and a right adrenal mass detected 2 weeks previously. His blood pressure was 200/110 mm Hg. B-ultrasound and CT revealed a right adrenal mass. Laparoscopic dissection of the right adrenal tumor was performed, and histopathological examination revealed right PCC. In August 2017, he was readmitted to the hospital after the detection of a left adrenal mass during a physical examination when he was 36 years old. His blood pressure fluctuated in the range of 120–130/75–90 mmHg, and his plasma norepinephrine concentration was elevated to 2,766.00ng/L (reference range, 0–600 ng/L). B-ultrasound and CT scanning revealed a 3.8 × 2.9 cm mass in the left adrenal gland. Biochemical and imaging examinations revealed no evidence of pituitary abnormalities or other related NET. The diagnosis was “left PCC after right PCC dissection.” After treatment with terazosin hydrochloride and volume expansion for 1 week, the patient underwent laparoscopic left adrenal tumor resection. The pathological results indicated a left PCC. The final diagnosis was a metachronous bilateral PCC. Pedigree investigations and genetic testing were performed in May 2020 (**Figures 1**-FA-I-1, I-2, II-1, III-1). Only the proband and his 13-year-old son (**Figures 1**-FA-III-1) carried a heterozygous nonsense mutation in exon 4 of *MAX* (p.R75*, c.223C>T; NM_002382.5; **Figure 2A**). Systematic screening of the pituitary gland, neck, chest, and abdomen showed no abnormalities.

#### 3.1.2 Family B

The proband (**Figures 1**-FB-II-1), a 34-year-old male, was admitted to the hospital in December 2021 with bilateral adrenal lesions. He had no symptoms, such as headache, palpitations, or excessive sweating. His blood pressure fluctuated in the range of 110–125/60–80 mmHg. B-ultrasound and CT examinations revealed bilateral adrenal masses (left, 2.8 cm; right, 2.2 cm). Plasma catecholamine, metanephrine, and normetanephrine levels were within normal limits. Imaging of the pituitary gland and neck and related biochemical examinations, such as serum calcium, calcitonin, parathyroid hormone, growth hormone, prolactin and so on, revealed no abnormalities. The patient was suspected to have “simultaneous bilateral PCC.” Subsequently, laparoscopic bilateral adrenal-sparing surgery was performed after treatment with terazosin hydrochloride and volume expansion for 1 week. Pathological results confirmed bilateral PCC. In January 2022, a family investigation and genetic testing (FB-II-1 and FB-II-1) were performed and the proband (FB-II-1) was found to carry a heterozygous frameshift mutation in exon 4 of *MAX* (p.Q82Tfs*89, c.242_243dupAC; NM_002382.5; **Figure 2B**). However, his parents (FB-I-1 and FB-I-2) showed no evidence of abnormalities in the pituitary gland, neck, chest, or abdomen. The proband refused genetic testing of his parents and his 3-year-old daughter (FB-III-1).

#### 3.1.3 Family C

The proband (**Figures 1**-FC-II-1), a 64-year-old female, was admitted to our hospital in November 2022 with right adrenal lesions. She occasionally complained of paroxysmal headache. Her blood pressure fluctuated in the range of 100–130/66–90 mmHg. B-ultrasound and CT scanning revealed a 1.9 × 1.6 cm mass in the right adrenal gland. The plasma norepinephrine concentration was elevated to 2,243.7pmol/L. Imaging and biochemical examinations showed no evidence of pituitary, neck, or chest abnormalities. The diagnosis was “suspicion of right PCC.” After phenoxybenzamine and volume expansion treatment for 1 week, the patient underwent laparoscopic right adrenalectomy. A pathological report revealed a right PCC. In March 2024, genetic testing revealed a heterozygous missense mutation in exon 5 of *MAX* in the proband (p.E158A, c.473A>C; NM_002382.5; (**Figure 2C**). The mutation was detected in only one of her daughters and one of her sisters (**Figures 1**-FC-II-3, III-1), but their adrenal CT examination and blood biochemistry, such as plasma catecholamine, metanephrine, and normetanephrine, and so on, showed no abnormalities.

### 3.2 *In silico* analyses and 3D structure of MAX variants

Six of 19 members of the three PPGL families with PCC harbored germline *MAX* variants (**Figure 1**, **Table 1**). Of these, only three (FA-II-1, FB-II-1, and FC-II-1) presented with PCC, including one with metachronous PCC, one with simultaneous bilateral PCC, and one with unilateral PCC. Their ages at the initial diagnosis were 24, 34, and 64 years, respectively. The remaining three (FA-III-1, and FC-II-3, FC-III-1), aged 13, 59, and 43 years, respectively, had no evidence of PPGL or clinical symptoms or biochemical or imaging findings of pituitary adenoma (PA) or other NET.

**Table 1:** Variation types and clinical characteristics of 87 MAX germline mutation-associated PPGL families.

*MAX* mutation (Nucleotide change)	*MAX* mutation *Families/*carriers	Sex (M/F)	PCC					PGL N (%) ^a^	PA		Other NET		Reference
			N (%).^a^	Age at onset^#^ $\left(\bar{x}\pm s\right)$	unilateral/bilateral (N)	N of metastasis (%) ^b^	Age at metastasis $\left(\bar{x}\pm s\right)$		N (%) ^a^	Pituitary hormone	N (%) ^a^	Histological type (N)	
p.R33* (c.97C>T)	19/23	16/7	22(95.6)	29.0 ± 10.0	11/11	6(27.3)	35.5 ± 12.9	2(8.7)	0	—	3(13.0)	Abdomen NB(1), CCH(1), Adrenal GN (1)	(10, 11, 13, 16, 18, 28, 33, 34)
p.R75* (c.223C>T)	11/15	4/10^▲^	14(93.3)	30.3 ± 6.8^▲^	3/11	1(7.1)	38	1(7.1)	1(7.1)	GH,PRL↑	1(7.1)	PHPT (1)	[ (10–13, 19, 30, 32), Current study]
p.A67D(c.200C>A)	1/8	6/2	6(75)	29.0 ± 13.0	1/5	2(33.3)	51/61	1(12.5)	1(12.5)	GH ↑	2(25)	GN (1), Abdomen NB (1)	(14)
p.= (c.-18C>T)	1/1	0/1	0	—	0	—	—	1(100)	0	—	0	—	(11)
c.1_171del	1/1	0/1	1(100)	37	1/0	0	—	0	0	—	—	—	(33)
p.M1?(c.1A>G)	2/3	2/1	3(100)	29/36/46	1/2	1(33.3)	46	0	0	—	—	—	(10, 13)
p.M1?(c.2T>A)	1/1	0/1	1(100)	46	0/1	0	—	1(100)	0	—	—	—	(11)
p.M1? (c.3G>A)	2/2	1/1	2(100)	33/39	0/2	1(50)	33	0	0	—	—	—	(26, 32)
p.E8*(c.22G>T)	1/1	0/1	1(100)	21	0/1	0	—	0	1(100)	PRL↑	1(100)	PHPT(1)	(14)
p.V9L (c.25G>T)	1/1	1/0	1(100)	13	1/0	0		1(100)	0	—	—	—	(11)
p.V9Wfs*56 (c.25del)	1/1	0/1	1(100)	56	1/0	0	—	0	0	—	—	—	(11)
p.= (c. 63G>T) ^$^	1/1	1/0	1(100)	22	1/0	0	—	0	0	—	—	—	(11)
p.D23N (c.67G>A)	1/1	0/1	1(100)	26	1/0	1(100)	26	0	0	—	—	—	(10)
p.K24fs*40 (c.70_73delAAAC)	1/1	0/1	1(100)	24	0/1	0	—	1(100)	0	—	—	—	(22)
p.R25W(c.73C>T)	2/2	0/2	2(100)	36/43	1/1	0	—	1(50)	0	—	—	—	(11, 12)
p.R35C (c.103C>T)	1/1	0/1	1(100)	57	1/0	0	—	0	0	—	—	—	(11)
p.R47_S52del (c.140_157del)	1/1	1/0	1(100)	24	1/0	0	—	0	0	—	—	—	(11)
p.S49*(c.146C>G)	1/1	0/1	1(100)	23	1/0	0	—	0	0	—	—	—	(12)
p.S52*(c.155C>G)	1/1	1/0	1(100)	43	0/1	0	—	0	0	—	—	—	(33)
p.Q54R(c.161T>A)	1/1	NA	1(100)	NA	0/1	0	—	0	0	—	—	—	(24)
p.R60*(c.178C>T)	1/1	0/1	1(100)	55	0/1	0	—	0	0	—	—	—	(11)
p.Q62Kfs*104(c.183_195del)	1/1	1/0	1(100)	20	0/1	1(100)	45	0	0	—	1(100)	PHPT(1)	(36)
p.Q62Nfs*23(c.185_186delA)	1/1	0/1	1(100)	47	0/1	0	—	0	0	—	—	—	(10)
p.R66* (c.196C>T)	1/1	NA	1(100)	NA	0/1	0	—	0	0	—	—	—	(24)
p.F67V(c.199A>C)	1/1	1/0	1(100)	59	1/0	0	—	0	0	—	—	—	(33)
p.I71S (c.212T>G)	1/1	0/1	1(100)	34	0/1	0	—	0	0	—	—	—	(11)
p.M74V(c.220A>G)	1/1	1/0	1(100)	57	1/0	1(100)	57	0	1(100)	NA	—	—	(11)
p.H81Pfs*5 (c.242_243del)	1/1	0/1	1(100)	50	0/1	0	—	0	0	—	—	—	(12)
p.Q82Tfs*89(c.242_243dupAC)	1/1	1/0	1(100)	32	0/1	0	—	0	0	—	—	—	Current study
p.E82*(c.244C>T)	1/1	0/1	1(100)	18	1/0	1(100)	18	0	0	—	—	—	(11)
p.Q91*(c.271C>T)	1/2	1/1	2(100)	26/32	0/2	0	—	0	0	—	—	—	(35)
p.L94P(c.281T>C)	1/1	0/1	1(100)	41	1/0	0	—	0	0	—	—	—	(10)
p.Q98Pfs*48(c.292dupT)	1/1	1/0	1(100)	21	1/0	0	—	0	0	—	—	—	(12)
p.R90P (c.269G>C)	1/1	0/1	1(100)	29	1/0	0	—	0	0	—	—	—	(11)
p.R100P(c.299G>C)	1/1	0/1	1(100)	15	0/1	0	—	1(100)	0	—	1(100)	GNB/Adrenal GN (1)	(27)
p.L102P(c.305T>C)	1/1	1/0	1(100)	13	1/0	0	—	0	0	—	—	—	(11)
p.E103*(c.307G>T)	1/1	1/0	1(100)	26	0/1	0	—	0	0	—	—	—	(12)
p.= c.414G>A^$^	1/1	0/1	1(100)	80	1/0	0	—	0	0	—	—	—	(11)
p.S142L(c.425C>T)	1/1	0/1	1(100)	22	1/0	0	—	0	0	—	—	—	(10)
p.E158A (c.473A>C)	1/3	0/3	1(33.3)	64	1/0	0	—	0	0	—	—	—	Current study
c.1-?_483+?del	1/1	1/0	1(100)	27	1/0	0	—	0	0	—	—	—	(11)
c.64-2A>G	1/1	0/1	1(100)	38	1/0	0	—	0	0	—	—	—	(29)
c.171 + 1G>A	2/2	0/2	2(100)	18/37	0/2	0	—	1(50)	0	—	—	—	(11, 23)
c.172-3C>G	1/1	1/0	1(100)	21	1/0	0	—	0	0	—	—	—	(33)
c.295 + 1G>A	2/2	2/0	2(100)	32/40	0/2	1(50)	32	0	0	—	—	—	(10, 31)
c.295 + 1G>T	1/1	0/1	1(100)	40	0/1	1(100)	40	1(100)	0	—	—	—	(11)
c.296-1G>T	1/1	0/1	1(100)	49	0/1	0	—	0	1(100)	PRL↑	1(100)	PHPT(1)	(21)
c.397-2A>G	1/1	1/0	1(100)	26	1/0	0	—	0	0	—	—	—	(17)
Exon 1, 2 del	1/1	NA	1(100)	NA	1/0	0	—	0	0	—	0	—	(32)
Intron 1, Exon 1-3 del	1/1	0/1	1(100)	35	0/1	0	—	0	1(100)	GH ↑	—	—	(15)
Exon 3 del	2/3	3/0	2(66.7)	32/32	2/0	0	—	0	2(66.7)	PRL↑	1(33.3)	Pancreas NET (1)	(15, 25)
Exon 4 del	1/1	1/0	1(100)	22	0/1	1(100)	33	0	1(100)	GH ↑		—	(15)
*MAX-FUT8* Fusion	1/3	3/0	3(100)	28/45/55	0/3	1(33.3)	28	0	0	—	—	—	(20)
**Total**	87/109	59/46^▲^	101(92.7)	32.8 ± 12.6^▲^	42/59	19(18.8)	38.7 ± 12.8^▲^	12(11.0)	9(8.3)		11(10.1)		

PPGL, Pheochromocytoma and Paraganglioma; PCC, Pheochromocytoma; PGL, Paraganglioma; N, Number of patients; PA, Pituitary adenoma; NET, Neuroendocrine tumor; GH, Growth hormone; PRL, Prolactin; NB, Neuroblastoma; CCH, C-cell hyperplasia; GN, Ganglioneuroma; PHPT, Primary hyperparathyroidism; GNB, Ganglioneuroblastoma; a, Proportion of PCC/PGL/PA occurrence in each genotype; b. Proportion of metastatic PCC in the overall PCC patients; ^#,^When the number of patients is less than 3, it is expressed by specific age; ^$,^Only synonymous mutations were found and remain to be clarified ^▲,^ Available data.

**Figure 3:**
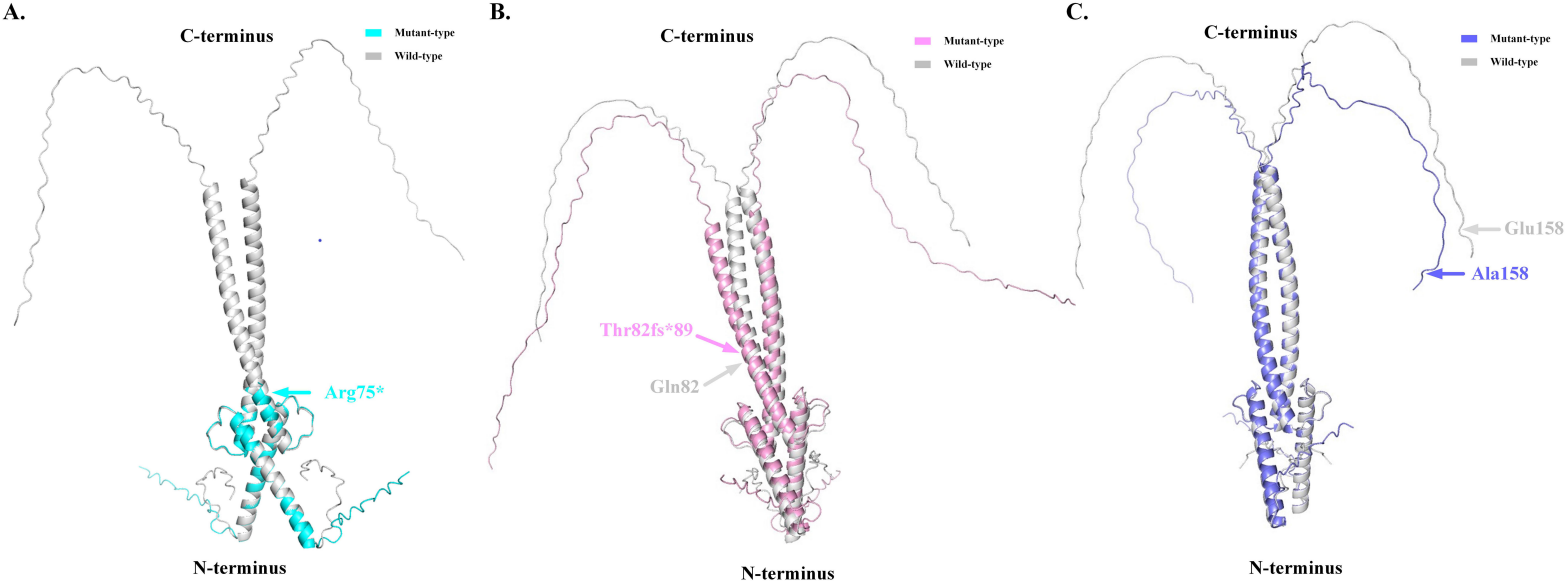
The predicted three-dimensional structure diagrams of the mutant-type MAX protein based on Alphafold 2 V2.3.1 (Bodies of grey represented as the wide type, and colored bodies represented as mutant types). **(A)**. p.R75* mutation leads to the truncation of MAX protein. **(B)**. Thr82fs89 alteration leads to the shortening of the C-terminal of MAX protein. **(C)**. p.E158A mutation leads to the alteration in the Loop domain of MAX protein.

The pathogenicity of the *MAX* variants was predicted using CADD and REVEL. High scores in the CADD (> 20) and REVEL (> 0.5; only evaluated for missense variants) implies a greater likelihood of the variant causing disease. The CADD values of the mutations c.223C>T and c.473A>C were 36 and 25, respectively, while the REVEL value of the mutation c.473A>C was 0.528, indicating that these two mutations are deleterious. Although the pathogenicity of the frameshift mutation c.242_243dupAC could not be predicted using the CADD and REVEL software, in combination with its clinical features, it is reasonable to consider it to be pathogenic (**Table 2**). In addition, the predicted 3D structure of the MAX protein indicated a structural change (**Figure 3**).

**Table 2:** The pathogenicity of *MAX* variants (NM_002382.5, hg38).

Gene	Chr	Variant	Nucleotide	Exon	Position	CADD	REVEL
*MAX*	14q23.3	p.R75*	c.223C>T	4	65544703	36	NA
		p.Q82Tfs*89	c.242_243dupAC	4	65544683-65544684	NA	NA
		p.E158A	c.473A>C	5	65543204	25.0	0.528

Chr, chromosome; CADD, Combined Annotation Dependent Depletion; REVEL, Rare Exome Variant Ensemble Learner; NA, not available.

### 3.3 Clustered data for patients with germline MAX mutations

The clinical presentations of the six patients from the three families we reported and the remaining 103 patients from 84 families reported in the literature are shown in **Table 1**. Of the 53 *MAX* germline alterations identified, the most common mutations were nonsense mutations (47.7%), followed by missense mutations (23.8%), large insertions or deletions (14.8%), intronic variants (9.2%), synonymous mutations (1.8%), and gene fusion mutations (2.7%). The three most common mutations were p.R33* (c.97C>T; 21.1%), p.R75* (c.223C>T; 13.8%), and p.A67D (c.200C>A; 7.3%). The mutation rates in exons 1 to exon 5 were 8.3%, 0.1%, 29.4%, 34.9%, and 7.3%, respectively. In addition, another 10.1% of the alterations involved multiple exon deletions, and 9.2% of the variations occurred in introns.

Overall, 101 (92.7%) of the 109 patients developed PCC, with a mean age at diagnosis of 32.8 ± 12.6 years (range, 13–80), and the male-to-female ratio was 1.3:1. Of these, 59 (58.4%) developed bilateral PCC, with a mean diagnostic age of 31.7 ± 10.2 years (range, 14–55 years), of which 47 had simultaneous PCC and 12 had metachronous bilateral PCC with a mean interval time of 11.1 ± 6.7 years (range, 3–27 years). The remaining 42 (41.6%) were unilateral PCC with a mean diagnostic age of 34.3 ± 15.4 years (range, 13–80). Nineteen (18.8%) of the patients were diagnosed with metastatic PCC. Eleven (10.9%) had concomitant chest or abdominal PGL, and one presented with head and neck PGL without PCC. Additionally, the detection of the levels of blood/urine catecholamines and their metabolites were also reported in 63 of the 101 patients with PCC. The results showed that 61 (96.8%) were positive, of which 18 (29.5%) were positive for an increase in a single normetanephrine/norepinephrine and 43 (70.5%) were positive for a mixed increase in metanephrine/adrenaline, normetanephrine/norepinephrine, and/or dopamine.

Overall, nine (8.3%) patients were complicated with pituitary NET (PitNET) (16, 20, 24, 27). Among them, three, four, and one patient had a single increase in growth hormone, prolactin, and both hormone and prolactin concentrations, respectively, and were diagnosed with growth hormone tumors, prolactinomas, and mixed adenomas, showing acromegaly and/or prolactinoma symptoms. The hormone levels in the other patient were not reported. Four patients were diagnosed with PCC first, three with PA first, and two with PCC and PA simultaneously. Eleven (10.1%) developed other NETs, with an age at diagnosis ranging from 0.7 to 60 years, including seven combined with PCC: four primary hyperparathyroidism(PHPT) cases and each of one case in neuroblastoma, pelvic ganglioneuroblastoma/ganglionoma, and C-cell hyperplasia. Therein, the four PHPT patients’ mean diagnosis age was 41.8 ± 17.8 years(range, 18-60). Among them, one case showed multifocal parathyroid adenomas on thyroid ultrasound and was confirmed by postoperative pathology which typical of hereditary diseases. One case showed a single solid-appearing lower pole nodule of the left lobe without suspicious malignant features but did not receive surgical treatment. The other two patients’ thyroid ultrasound were not reported. In seven (6.4%) patients, PCC was accompanied by non-NETs, including one each of squamous cell carcinoma of the tongue, papillary thyroid carcinoma, prostate cancer, renal cell carcinoma, renal oncocytoma, breast cancer combined with renal oncocytoma, and chondrosarcoma of the chest combined with multifocal lung adenocarcinoma. In addition, eight (7.3%) patients with *MAX* mutations did not have PCC, but two had ganglioneomas, one had abdominal ganglioneuroblastoma, one had pancreatic NET, one head and neck PGL, and three were asymptomatic carriers. Among the 87 families, 22 (25.3%) had a family history of PPGL and eight (9.2%) had a clear paternal family genetic background. The ages at diagnosis of the different patient types are shown in **Table 3**. The occurrence rates of PCC, metastatic PCC, PGL, PA, other NET, and non-NET for the different mutation types (groups) are shown in **Table 4**. There was no significant difference in the age at diagnosis or the occurrence rate between Groups (all *P* > 0.05).

**Table 3:** The diagnosis age of different types (Groups) of patients.

	The diagnosis age (years)	*P* value
Patients with PCC metastasis	32.6 ± 12.4	0.963
Patients without PCC metastasis	32.8 ± 12.8	
Patients with PCC and PA		
Presence of PCC	32.1 ± 13.3	0.535
Presence of PA	34.6 ± 14.3	
PCC patients with PA	32.1 ± 13.3	0.874
PCC patients without PA	32.8 ± 12.6	
Patients with GM	32.3 ± 18.5	0.990
Patients with GN	32.3 ± 11.3	

PCC, Pheochromocytoma; PGL, Paraganglioma; PA, Pituitary adenoma. GM, Group missense mutations; GN,Group non-missense mutations.

**Table 4:** The phenotypes’ occurrence rates of different mutation Groups.

	Occurrence rate (%)		*P* value
	Patients with GM	Patients with GN	
PCC	84.6	95.2	0.170
Metastasis PCC	15.4	18.1	0.985
PGL	15.4	9.6	0.647
PA	7.7	8.4	0.905
Other-NET	11.5	9.6	0.779
Non-NET	7.7	6.0	0.762

GM, Group missense mutations; GN, Group non-missense mutations; PCC, Pheochromocytoma; PGL, Paraganglioma; PA, Pituitary adenoma; NET, Neuroendocrine tumor.

## 4 Discussion

**Figure 4:**
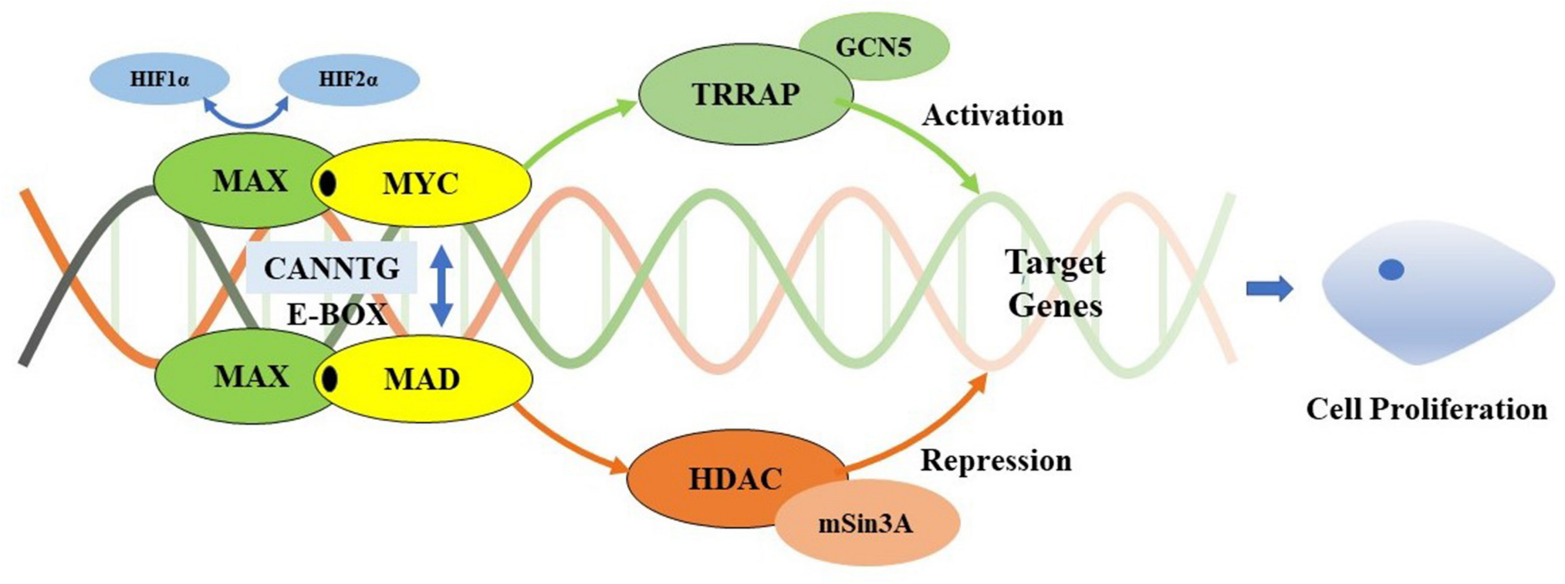
Myc/Max/Mad transcription module. MAX forms a specific DNA-binding protein complex with MYC or MAD which recognizes the core sequence 5’-CAC[GA] TG-3’(E-box). The MYC/MAX complex is a transcriptional activator, interacts with TRRAP that recruits the GCN5, which permitting transcription of target genes affecting cell proliferation. It can be regulated by HIF1α and HIF2α. HIF1α dimerizes with MAX and suppresses the binding to the E-box, while HIF2α leads to stabilization of the MYC/MAX complex to promote the activation of MYC target genes. Meanwhile, the MAD/MAX complex is a repressor which repress transcription by recruiting HDAC’s via mSin3A, and represses MYC transcriptional activity from E-box elements. MAX mutations result in preventing MYC-MAX heterodimerization and MAX-MAD homodimerization, inhibiting the suppression of E-boxs target DNA sequences transcription activity. However, pathogenic MAX mutations repress to a lesser extent MYC’s E-box binding ability. Increased expression of Myc appears to contribute to a more aggressive phenotype of chromaffin cells which develop from neural crest cells.

The *MAX* germline mutations were first reported to be associated with hereditary PPGL in 2011 (10). The prevalence of *MAX* mutations in PPGL varies from 0.8%–1.9% (11–13). To date, almost all *MAX* mutations in PPGL have been reported in case reports or small family-based cohorts. In this study, we report six patients from three families with p.R75*, p.Q82Tfs*89, and p.E158A *MAX* mutations, the latter two of which have never been previously reported. Among them, two presented with bilateral PCC and one presented with unilateral PCC, whereas the other three were asymptomatic carriers without any signs of NET. Based on a literature review (10–36), 87 families with 109 *MAX* mutations have been discovered, with 53 different *MAX* mutation types involving all five exons. Most mutations were located in exons 3 and 4 of *MAX*. The mutation types were mainly nonsense, missense, and alternative splice-site mutations, with nonsense mutations p.R33 (21.1%) and p.R75* (13.8%) and the missense mutation p.A67D (7.3%) were the most common mutations, suggesting that there may be family and/or regional aggregation of *MAX* mutations (10–14). The *MAX* gene, located on chromosome 14q23.3, encodes a 160-amino acid *MAX* protein containing an N-terminal basic helix-loop-helix leucine zipper (bHLHLZip) domain and six C-terminal tyrosinase kinase II phosphorylation sites. *MAX* forms a specific DNA-binding protein complex with MYC or MAD, which recognize the core sequence 5’-CAC[GA]TG-3′ (E-box). *MAX* mutations result in an abnormal structure of the bHLHLZip domain, preventing MYC-MAX heterodimerization and MAX-MAD homodimerization, and inhibiting the suppression of E-box target DNA sequence transcription activity and/or somatic-level MAX allele loss of heterozygosity (LOH) (37, 38). However, pathogenic *MAX* mutations repressed the E-box-binding ability of MYC to a lesser extent (**Figure 4**) (39). Increased MYC expression levels appear to contribute to a more aggressive phenotype of chromaffin cells that develop from neural crest cells (40). MAX had a loss-of-function intolerance probability (pLI) score from the Genome Aggregation Database of 0.83, which was close to 1. A higher pLI score indicates that the gene is more intolerant to loss-of-function, implying that the mutation is more likely to be harmful. This explains the high pathogenicity and penetrance of *MAX* mutations. Although the *MAX* mutation is classified as type C2, including *RET, NF1, TMEM127,H-RAS* and *ATRX*, which belongs to the SWI/SNF family of chromatin remodeling proteins, as their upregulation will activate the PI3K/AKT and RAS/MAPK signaling pathways resulting in tumor formation (41). It’s clinical phenotype is similar to *SDHD-* and *SDHAF2*-related PPGL (C1 type), which is associated with metastatic PPGL and its underlying mechanism involves the “Warburg effect”, which means that genetic mutations lead to damage to aerobic glycolysis and oxidative phosphorylation functions, as well as the mechanism of the HIF-MYC/MAX interaction (1, 2).

In all, 101 out of the 109 patients (92.7%) with *MAX* mutations developed PCC, of which bilateral PCC accounted for 58.4%, with a mean age at diagnosis of 31.7 ± 10.2 years (range, 14–55). Different from other classic PPGL genetic syndromes, which have a PCC incidence rate of 1%–50% (1, 2, 9), as well as sporadic PCC, which often occurs unilaterally, with an average age at diagnosis greater than 45 years (1, 9), *MAX* mutations always lead to a high incidence of bilateral PPGL and early onset In this study, 19 (18.8%) patients were diagnosed with metastatic PCC at the time of diagnosis, and the youngest age at diagnosis was 18 years, suggesting that patients with *MAX* mutations had a higher metastatic risk and a younger age at diagnosis than patients with non-metastatic PCC, although the difference was not statistically significant (*P* = 0.963). The PCC/PGL phenotype is most commonly observed in *VHL-, FH-*, and *HIF2A/EPAS1*-related syndromes (1, 2, 9, 14). In this study, 11 (10.9%) patients had PCC combined with thoracic or abdominal PGL, and the youngest age at diagnosis was 15 years. One other patient only presented with head and neck PGL, without PCC. This may be a characteristic manifestation of *MAX* mutations or it may be associated with the latent onset of PGL in the head and neck, which is often asymptomatic and can be easily misdiagnosed. Available data also suggest that *MAX* mutations are mostly characterized by a mixed phenotype of noradrenaline/adrenaline secretion rather than a single adrenaline secretion pattern (11, 14–23, 27–33), which may be related to *MAX* mutations leading to a decrease in phenylethanolamine N-methyltransferase expression levels, resulting in weakened conversion of noradrenaline to adrenaline (38). The positive rate of detecting blood/urine catecholamines and their metabolites metanephrine/normetanephrine in patients with *MAX*-mutation-related PPGL was 96.8% (11, 14–23, 26–35), which could benefit the preoperative qualitative diagnosis of such types of PPGL (1, 8, 9, 42).

Nine (8.3%) patients had PA, with the youngest age at diagnosis of 16 years. Most patients showed elevated levels of growth hormone only (3/9), prolactin only (4/9), or both (1/9). They manifested clinically as acromegaly and prolactinomas (14, 15, 21, 25, 30). The age at diagnosis was later for PA than for PCC, but the difference was not statistically significant (*P* = 0.535). PPGL with PA is also known as “3PA syndrome”, and was originally reported to be associated with pathogenic *SDHA-D* mutations, which was the most common pathogenic cause of familial 3PA syndrome (62.5% to 75%) (14, 43). Considering that the prevalence of PA is approximately 1/3,000, the high frequency of PitNET in *MAX* mutation carriers is not coincidental. *MAX* is a pathogenic susceptibility gene, although the direct mechanism of *MAX* in related PA remains to be elucidated (14).

Eleven (10.1%) patients had other NETs, diagnosed between 0.7 and 60 years old, including seven cases combined with PCC (14, 25, 28). Considering that these tumors (four parathyroid adenoma and one each of neuroblastoma, ganglioneuroblastoma/ganglionoma, and C-cell hyperplasia), PPGL, and PitNETs all originate from embryonic neural crest cells on histological examination, it is reasonable to assume that *MAX* mutations may trigger such tumors. In a cohort study of 372 neuroblastomas, it was found that 22% of tumor cells had LOH at chromosome 14q, especially at 14q23, including *MAX*. This further supports the close relationship between *MAX* mutations and the occurrence of these tumors (14, 44). The other four patients (3.7%) had single occurrences of ganglioneuroma (n = 2), abdominal neuroblastoma (n = 1), and pancreatic NET (n = 1) with the youngest age at diagnosis of 0.7 years (14, 25, 28). It is suspected that ancestral cells may overlap and have different expression spectra, and mature PPGL (type C2) originates from the chromaffin cell lineage, while mature neuroblastoma originates from the sympathetic neuroblastoma lineage. It may imply that *MAX* mutants have undergone a two-hit of single tumor stem cells on their second chromosome early in embryogenesis, before neural crest cells migrate and localize to the paraganglia or adrenal glands (39, 40). Two-hit hypothesis holds that certain genes (such as tumor suppressor genes) need to undergo “two hits”, that is, both alleles mutate or are inactivated, before tumors will occur. In hereditary tumors, the first hit is usually a germline variation of a certain tumor suppressor gene in germ cells, making the individual genetically susceptible; the second hit is a somatic variation of the other allele of this gene after birth, thereby promoting the occurrence and development of tumors. The *MAX* gene belongs to tumor suppressor genes, and its mutations may also follow the “two-hit” hypothesis (39, 40).

Seven (6.4%) patients had non-NETs (11, 14, 15, 20). The small number of cases and the involvement of six different systems and tissues (tongue, thyroid, kidney, prostate, breast, cartilage, and lung) may be an incidental or concomitant phenomenon; however, it is still difficult to exclude the possibility of a possible association with the *MAX* mutation. It should be noted, however, that of 109 patients with *MAX* mutations, three (2.8%) still had no evidence of a clinical phenotype.

Using *MAX* mutation genotype-phenotype analysis, we compared the occurrence rate of PCC (*P* = 0.170), metastatic PCC (*P* = 0.985), PGL (*P* = 0.647), PA (*P* = 0.905), other-NET (*P* = 0.779), and non-NET (*P* = 0.762), and the mean age at diagnosis at the presentation of PCC (*P* = 0.990) between patients with missense and non-missense mutations. The results indicated that the high penetrance of *MAX* mutation-related PCC and relatively high metastatic risk may not be related to *MAX* mutation types, or that the genotype-phenotype correlation is not strong. In addition, only 22 (25.3%) of the 87 *MAX* mutation pedigrees had a clear family history of PPGL (10–14, 20, 25–29, 33–35), similar to the case for *RET* mutations causing MEN2B (type C2) (42). Including the patient in pedigree II-1 of family A, *MAX* mutations may also have a very high *de novo* mutation rate (65.8%), and *de novo MAX* mutations may be a common pathogenic form. equations should be inserted in editable format from the equation editor.

## 5 Conclusions

In conclusion, *MAX* germline mutations may cause a new type of MEN originating from the neural crest, which may be called MEN 5 (14, 25, 45, 46). This suggests that all *MAX* germline mutation carriers should receive a comprehensive baseline assessment of the diversity of neurocrest-derived diseases, such as PPGL, PA, hyperparathyroidism, and ganglioneuroma/neuroblastoma. The appropriate frequency (interval) and extent of evaluation are optimized, but should take into consideration the risk preferences for bilateral and/or metastatic PCC, with lifelong intensive surveillance. Meanwhile, for patients with new diagnoses of PPGL, NGS is recommended to perform to identify potential mutations in all known susceptibility genes.

## Data Availability

The original contributions presented in the study are included in the article/supplementary material. Further inquiries can be directed to the corresponding authors.
